# Comparative Analysis of the Metabolome and Transcriptome between the Green and Yellow-Green Regions of Variegated Leaves in a Mutant Variety of the Tree Species *Pteroceltis tatarinowii*

**DOI:** 10.3390/ijms23094950

**Published:** 2022-04-29

**Authors:** Qian Qiao, Chong Wu, Tian-Tian Cheng, Yu Yan, Lin Zhang, Ying-Lin Wan, Jia-Wei Wang, Qing-Zhong Liu, Zhen Feng, Yan Liu

**Affiliations:** 1 Beijing Key Laboratory of Ornamental Plants Germplasm Innovation & Molecular Breeding, National Engineering Research Center for Floriculture, Beijing Laboratory of Urban and Rural Ecological Environment, Key Laboratory of Genetics and Breeding in Forest Trees and Ornamental Plants of Ministry of Education, School of Landscape Architecture, Beijing Forestry University, Beijing 100083, China; qiaoq404@bjfu.edu.cn (Q.Q.); wan_yingling@bjfu.edu.cn (Y.-L.W.); 2 Shandong Institute of Pomology, Shandong Academy of Agricultural Sciences, Tai’an 271000, China; wuchongge@gmail.com (C.W.); wangjwsdip@gmail.com (J.-W.W.); qzliu001@gmail.com (Q.-Z.L.); 3 Taishan Forestry Science Institute, Tai’an 271018, China; Chengtiantian@ta.shandong.cn (T.-T.C.); lkyyanyu@gmail.com (Y.Y.); lkyzhanglin@gmail.com (L.Z.); 4 Department of Forestry, College of Forestry, Shandong Agricultural University, Tai’an 271018, China

**Keywords:** *Pteroceltis tatarinowii*, variegated, transmission electron microscopy, transcriptome, metabolism, combined analysis

## Abstract

In nature, many different factors cause plants to develop variegated leaves. To explore the mechanism of variegated leaf formation in *Pteroceltis tatarinowii*, a mutant variety (‘Jinyuyuan’), which was induced by ethylmethylsulfone, was selected, and its morphological structure, physiology, biochemistry, transcription and metabolism were analysed. According to differences in colour values, the colours were divided into two regions: a green region and a yellow-green region. The chlorophyll content of the two regions was significantly different. Moreover, the yellow-green regions of the leaves were significantly thinner than the green regions. The chloroplast ultrastructure in the yellow-green region revealed small chloroplasts, large vacuoles, small starch grains, obviously increased numbers of osmophilic grains, loose lamellae of the inner capsule and thin lamellae. Moreover, the yellow-green region was accompanied by oxidative stress, and the activity of the oxidative phosphorylation pathway related to oxidative activity in the transcriptome showed an upward trend. Vitamin B6 and proline contents also increased, indicating that the antioxidant activity of cells in the yellow-green region increased. Transcriptomic and metabolomic analysis showed that the differentially expressed genes (DEGs) related to chlorophyll synthesis and metabolism led to a decrease in the photosynthesis and then a decrease in the assimilation ability and contents of sucrose, starch and other assimilates. Amino acid synthesis and metabolism, lipid synthesis and the activity of metabolic pathways were obviously downregulated, and the contents of differentially accumulated metabolites associated with amino acids and lipids were also reduced. At the same time, 31 out of 32 DEGs involved in the flavonoid synthesis pathway were downregulated, which affected leaf colour. We hypothesized that the variegated leaves of *P. tatarinowii* ‘Jinyuyuan’ are caused by transcriptional and post-transcriptional regulation. Mutations in pigment and flavonoid synthesis pathway genes and transcription factor genes directly affect both pigment and flavonoid synthesis and degradation rate, which in turn affect carbon assimilation, carbon fixation, related protein synthesis and enzyme activity, lipid synthesis and degradation and the activity of other metabolic pathways, eventually leading to the formation of different colour regions.

## 1. Introduction

Variegated leaf plants are the general term for plants that have more than two colours on the same leaf, such as white, yellow, red and purple, including multicoloured flecks or veins. The colour changes in these plants differ from that of plants that change colour due to seasonal factors, and the spots and stripes of the former remain throughout their entire life [1]. The mechanism underlying this phenomenon involves differences in pigments in leaf cells. When chloroplasts are absent or abnormal, the chlorophyll production mechanism is hindered and dysregulated; gene mutations in the cells can occur; and the contents of anthocyanins, lutein and carotenoids increase. As a result, the leaves present different colours [2,3]. Studies on the occurrence, the genetic factors and the mechanisms of leaf colour variation of leaf colour mutants of model plant species such as *Arabidopsis thaliana* [4], rice [5], barley [6] and tobacco [7] have been carried out. Previous studies have shown that the nuclear genes *im* (*immutans*), *var1* (*variegated 1*), *var2* (*variegated 2*), *var3* (*variegated 3*), *thf1* (*thylakoid formation1*) and *chm/msh1* (*chloroplast mutator/muts homolog 1*); double mutations in *why 1* (*whirly 1*), *why3* (*whirly 3*), *msl 2* (*mechanosention-like proteins 2*) and *msl3* (*mechanosention-like proteins 3*); and mutations in the *atd2 (glutamine 5-phosphoribosylpyrophosphate amidotransferase 2-deficient)*, *cue1* (*CAB underexpression*) and *PAC* (*pale cress*) genes can all cause variegated phenotypes. In addition to nuclear gene mutations, some chloroplast and mitochondrial gene mutations can lead to leaf spot formation [8]. Based on the *var1*, *var2* and *im* genes of the model plant species *Arabidopsis thaliana* [9], three classic variegated leaf formation hypothesis models have been proposed. Namely, the FtsH threshold hypothesis, the protein synthesis and degradation hypothesis and the photosynthetic redox imbalance hypothesis have been considered. The FtsH threshold hypothesis suggests that FtsH deficiency impedes chloroplast development and ultimately leads to variegated leaf formation [10,11], the hypothesis of which is that there is a balance between protein synthesis and degradation based on the FtsH threshold model, which proposes that decreased chloroplast translational activity can inhibit leaf pigment formation [12,13]. The photosynthetic redox imbalance hypothesis suggests that excessive reduction in plastoquinone (PQ) is the main cause of chlorophyll photooxidation and the formation of white/yellow leaves [14]. Subsequently, it was proposed that the MEP pathway regulates isoprene synthesis, which, given that isoprene acts upstream of chlorophyll and carotenoid synthesis, may also play an important role in regulating leaf colour formation [15]. However, the molecular mechanism of leaf spot formation is still unclear [14,16,17], and it is very difficult to elucidate the mechanism of plant variegation. For example, it is unknown how mutant cells develop into yellow/white regions or green regions at the early stage of plastid differentiation and how the growth environment and physiological environment at a specific stage of plant development determine the formation of mosaic colour patterns in the same genetic background. The mechanism of how leaves differentiate into green and white or yellow patches and why normal and abnormal mutant colours are present in the same leaf remain unclear.

In recent years, studies on ornamental plants with spotted leaves have been performed; the plants of these studies include *Clivia miniata* var. *variegata* [18], *Hosta* ‘Gold Standard’ [19], *Cymbidium sinense* ‘Dharma’ [20], *Hydrangea macrophylla* ‘Maculata’ [3], *Aucuba japonica* ‘Variegata’ [21], *Ginkgo biloba* variegated leaf mutant [22], *Camellia sinensis* variegated leaf mutant [23], *Actinidia kolomikta* white and pink variegated leaf mutants [24], *Catalpa fargesii* ‘Maiyuanjinse’ [25], etc. Leaf spot formation in non-model plant species have some similarities and characteristics with that of model plant species. In addition, genes that are similar to those in model plant species have been isolated and identified in ornamental plant species, and these ornamental species may have genetic backgrounds significantly different from those of model species. Taken together, these findings improve the understanding of the mechanism underlying the variegated phenotype formation in plants.

*Pteroceltis tatarinowii* Maxim, the lone deciduous tree species of the genus *Pteroceltis* in the Ulmaceae family, is listed as a Class III national key protection object in the *Red Book of Chinese Plants*. It is a unique fibrous tree species in China and an important indicator of calcareous soil. It is not only an economically important tree species but also an important landscape tree species [26]. In this study, the differences in physiological, microstructural, transcriptional and metabolic elements in different leaf colour regions of the *P. tatarinowii* ‘Jinyuyuan’ mutant obtained by ethylmethylsulfone mutagenesis were measured and analysed to determine the cause of variegated leaf formation. Specifically, we sought to identify key differentially expressed genes (DEGs) and further improve the understanding of the mechanism of variegated leaf formation in plants. Our results also provide a theoretical basis both for analysing leaf colour variation in depth and for elucidating the molecular mechanism of leaf colour and formation and lay a foundation for the improvement of leaf colour ornamental traits via genetic engineering in the future.

## 2. Results

### 2.1. Morphological Observations

**Figure 1:**
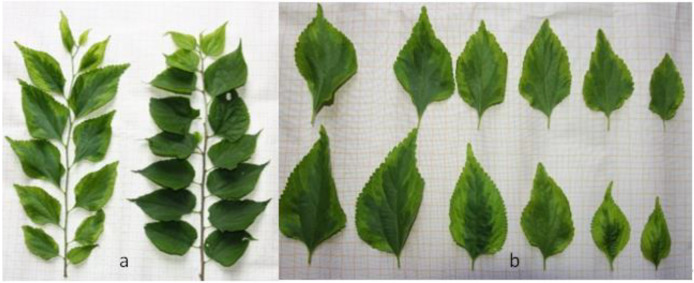
Shape of *P. tatarinowii* ‘Jinyuyuan’ leaves. (**a**) Comparison of *P. tatarinowii* ‘Jinyuyuan’ and wild-type plants; (**b**) details of the variegated leaves as they became larger.

The leaves of ‘Jinyuyuan’ could be divided into two regions according to differences in leaf colour, the characteristics of which are obviously different from those of the wild-type leaves (Figure 1a). With the growth of leaves, the green region (GR) tended to expand, while the area of the yellow-green region (YG) gradually decreased (Figure 1b). By comparing the leaf regions to the Royal Horticultural Society Colorimetric Card (RHSCC), we found that the green area was equal to 137B (belonging to the green group in RHSCC), and the yellow-green area was equal to N144D (belonging to the yellow-green group in RHSCC). The comparative analysis results of the chroma-meter show that GR and YG have very significant differences in terms of L, b and h (Table 1), indicating that the two are obviously different in colour. Compared with GR, YG has a brighter chromaticity and a more yellow colour.

### 2.2. Microstructure and Ultramicrostructure Observations

**Figure 2:**
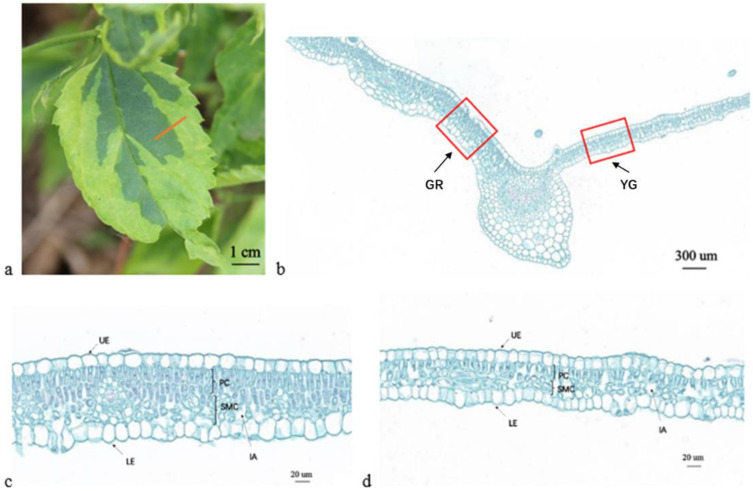
Microscopy observations of paraffin sections of *P. tatarinowii* ‘Jinyuyuan’ leaves. (**a**) *P. tatarinowii* ‘Jinyuyuan’ leaf. The red line indicates the cross-section position of the paraffin section. The green and yellow-green regions are separated along the main vein. (**b**) Microscopy observations of paraffin sections of *P. tatarinowii* ‘Jinyuyuan’ leaves. The red box is enlarged and displayed in (**c**,**d**). (**c**) Paraffin sections of the green region (GR). (**d**) Paraffin sections of the yellow-green region (YG). UE: upper epidermis, LE: lower epidermis, PC: palisade cells, SMC: spongy mesophyll cells, IA: intercellular air space.

Analysis of the paraffin section measurements showed that the thicknesses of the upper epidermis, lower epidermis, spongy tissue, palisade tissue and total area of the GR were greater than those of the YG (Figure 2). Among them, the total leaf thickness of GR was significantly greater than that of YG (Figure 2b–d), and there was a very significant difference between the two regions (Table 2). In addition, there were significant differences in spongy tissues, while there were no significant differences in the other indexes. According to Figure 2c,d, at the same magnification, the cells of the upper and lower epidermis, palisade tissue and spongy tissue in the green area were significantly larger than those in the yellow-green area, and the cells in the green area were closely arranged, while those in the yellow-green area were loose.

**Figure 3:**
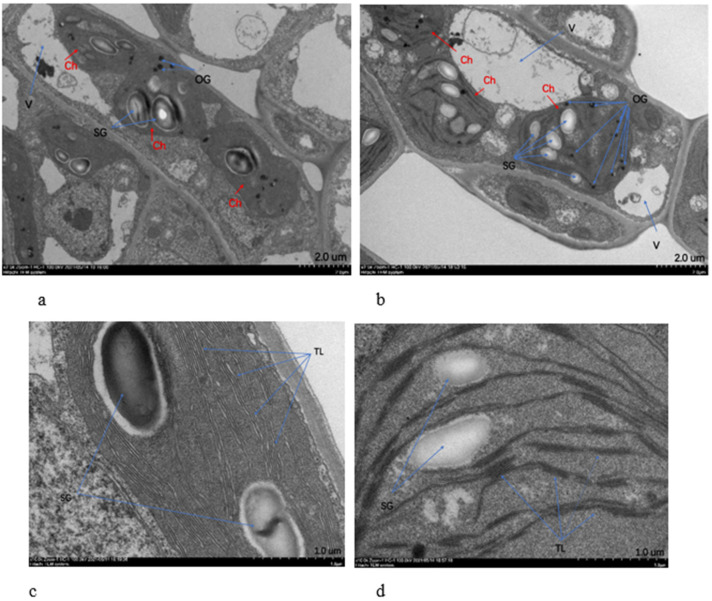
TEM images of *P. tatarinowii* ‘Jinyuyuan’. (**a**) TEM observation of cells in the green cells of leaves (GR). (**b**) TEM observation of yellow-green cells of leaves (YG). (**c**) Structure of the thylakoid lamellae in the green region of the leaf (GR). (**d**) Structure of the thylakoid lamellae in the yellow-green region of the leaf (YG). Ch: chloroplast, SG: starch granule, V: vacuole, OG: osmium granule, TL: thylakoid lamella.

According to the chloroplast transmission electron microscopy (TEM) images (Figure 3), the chloroplast structure in both regions was complete. The chloroplasts of the GR were long, oval or fusiform; present in large numbers; and had a large volume, occupying almost the whole cell. The vacuoles were small in volume. The starch grains were large in volume but were low in abundance (2–4), and osmium granules were rare (5–7) (Figure 3a). The YG chloroplasts were mostly ovoid, with a low abundance and small volume, accounting for a relatively small proportion of the cells. However, the vacuoles were large in volume. The total number of starch grains was large, although they were small, with more than 8–10 osmophilic granules (Figure 3b). Enlargement of the thylakoid structure showed that the thylakoid lamellae of the GR are neatly arranged and thick (Figure 3c), and YG lamellae are loosely arranged and thin (Figure 3d).

### 2.3. Determination of Pigment Contents

The content of chlorophyll in ‘Jinyuyuan’ leaves was significantly higher than that of carotenoids, which play a major role in leaf colour change, and the content of chlorophyll a was approximately twice that of chlorophyll b. All six pigment indexes were greater for GR than for YG, with significant or extremely significant differences (Table 3). These results indicated that there was a low chlorophyll content in YG.

### 2.4. Assays of Protective Enzyme Activities and MDA Contents

**Figure 4:**
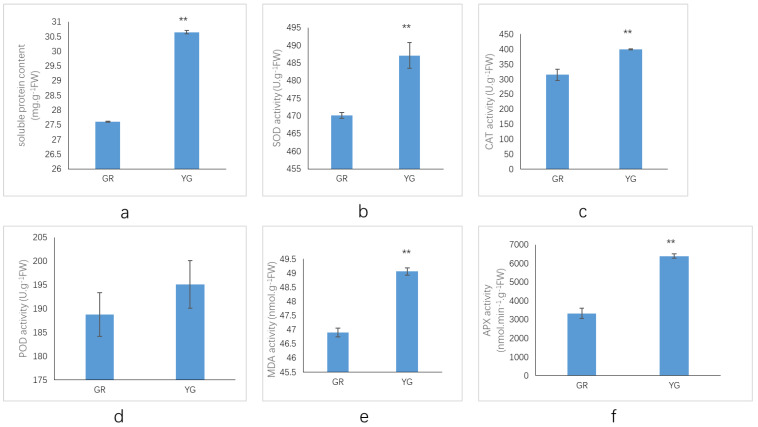
Activities of protective enzymes and MDA contents in green and yellow-green region of leaves of *P. tatarinowii* ‘Jinyuyuan’. (**a**) soluble protein content. (**b**) SOD activity. (**c**) CAT activity. (**d**) POD activity. (**e**) MDA activity. (**f**) APX activity. ** Indicates significance at the 0.01 level. GR indicates for the green region of the leaf, and YG indicates the yellow-green region of the leaf. A unit (U) is defined as giving 50% inhibition. APX activity unit definition: one enzyme activity unit oxidized 1 nmol AsA per g of tissue per minute.

Cellular oxidative damage is aggravated by excess reactive oxygen species (ROS). Activities of protective enzymes and malondialdehyde (MDA) contents were measured in the green and yellow-green zones of leaves to determine oxidative stress levels. According to Figure 4, the activities of superoxide dismutase (SOD), catalase (CAT) and ascorbate peroxidase (APX) were significantly enhanced in the yellow-green zone of leaves, which indicates higher activities of scavenging ROS. Moreover, the soluble protein content was significantly increased, which improves the water retention ability of cells and plays a protective role in cell biological substances and membranes. On the other hand, the MDA content was much higher in the yellow-green zones of leaves, which indicated more severe membrane-lipid peroxidation.

### 2.5. RNA-Seq and Assembly Analyses

#### 2.5.1. Quality Determination and Analysis of RNA Samples from Transcriptome Sequencing

A total of 39.5 Gb of clean data were obtained in this study, and more than 6.06 Gb of clean data of each sample were obtained. The Q20 base percentage was more than 98.14%, and the Q30 base percentage was more than 94.26%. The GC content ranged from 44.70% to 45.52% (Table S1).

**Figure 5:**
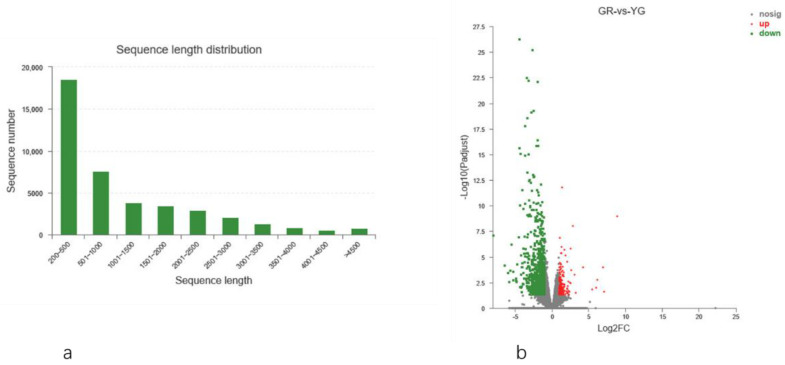
(**a**) Length distribution of transcripts; (**b**) volcano plot of DEGs. GR stand for the green region of the leaf and YG stands for the yellow-green region of the leaf.

A total of 41,833 unigenes and 80,323 transcripts were assembled, and the average N50 lengths were 2154 bp and 2161 bp, respectively. The average lengths of unigenes and transcripts were 1146.01 bp and 1424.1, respectively (Table S2). The length of most transcripts was 200–1000 bp, accounting for 62%, among which 200–500 bp fragments were the most abundant (18,557), accounting for 44% of the total number of unigenes (Figure 5a). These data indicated that the quality of the transcriptome sequencing data assembly was high, which was in line with the subsequent analysis results.

The assembled transcriptome sequences were compared against the contents of six databases (GO: Gene Ontology; KEGG: Kyoto Encyclopedia of Genes and Genomes; COG: Cluster of Orthologous Groups of proteins; NR: NCBI non-redundant protein sequences; Swiss-Prot: a manually annotated and reviewed protein sequence database; Pfam: Protein families databases), and a total of 22,926 genes were annotated, accounting for 54.80% of the total unigenes (Table S3). Among them, the unigenes had the most annotations in the NR database (22,701), accounting for 54.27% of the total number. This was followed by the GO database (19,002, 45.42%) and COG (18,257, 43.64%) database.

#### 2.5.2. Differentially Expressed Gene (DEG) Analysis

DEGseq2 software was used for filtering and analysing the DEGs, and filter conditions of |log_2_^Fold Change^| ≧ 1 and FDR < 0.05 were used. A total of 1169 genes were differentially expressed between GR and YG: 992 genes were downregulated, and 177 genes were upregulated. There were significantly more downregulated genes than upregulated genes (Figure 5b).

#### 2.5.3. GO Classification and Enrichment Analysis of DEGs

**Figure 6:**
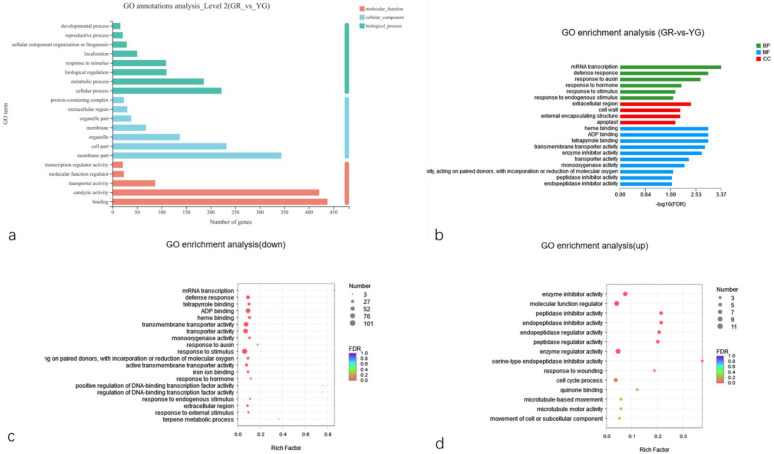
GO functional annotation and enrichment of DEGs. (**a**) GO functional annotation of DEGs. (**b**) GO enrichment of DEGs. (**c**) GO enrichment of downregulated DEGs. (**d**) GO enrichment of upregulated DEGs. GR indicates the green region of the leaf, and YG indicates the yellow-green region of the leaf.

To analyse the specific functions of the selected DEGs and identify the key genes related to the leaf colour differences, we first carried out GO secondary classification and enrichment analysis on the 1169 DEGs identified in the previous step (Figure 6a,b). The results of secondary classification showed that the DEGs were extremely significantly enriched in 20 GO terms, namely 8 secondary terms in the biological process (BP) category, 7 secondary terms in the cellular component (CC) category and 5 secondary terms in the molecular function (MF) category. Among them, protein binding and catalytic activity terms in the MF category, membrane part and cell part in the CC category, and cellular process and metabolic process in the BP category were associated with the most DEGs. The downregulated genes were mainly enriched in the defence response, the response to stimuli, the response to external stimuli, the response to hormones, response to auxin, ADP binding and haem binding (Figure 6c), indicating that the expression of YG’s DEGs in response to external stimuli, defence response and plant hormones decreased. There were significantly fewer upregulated gene GO enrichment pathways and DEGs than downregulated ones. The genes were mainly enriched in enzyme regulator activity, peptidase regulator activity, enzyme inhibitor activity, peptidase inhibitor activity, endopeptidase inhibitor activity, serine-type endopeptidase inhibitor activity and other enzyme-related pathways (Figure 6d). Taken together, these results showed that the expression of YG responsive enzyme-related genes increased.

#### 2.5.4. KEGG Classification and Enrichment Analysis of DEGs

**Figure 7:**
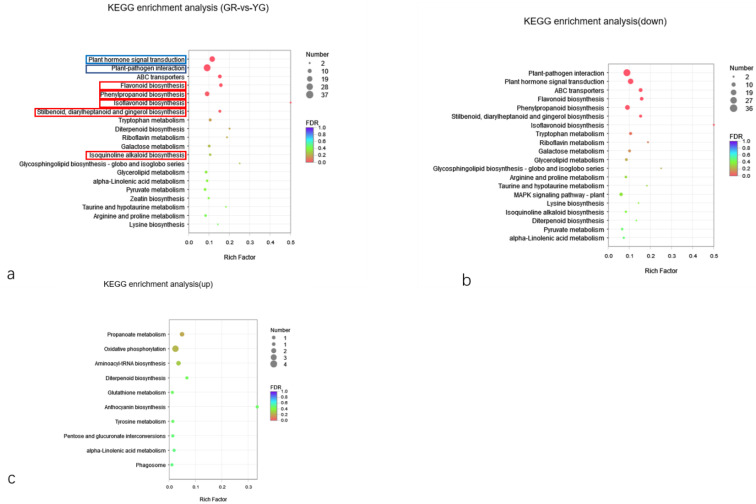
Scatter plot of enrichment factors in the KEGG pathways of DEGs. (**a**) KEGG enrichment bubble map. The blue box indicates the response to external stimuli and plant hormone signal transduction. The red box indicates the pathway of flavonoid synthesis. (**b**) KEGG enrichment bubble map of downregulated DEGs. (**c**) KEGG enrichment bubble map of upregulated DEGs. GR indicates the green region of the leaf, and YG indicates the yellow-green region of the leaf.

KEGG pathway classification and enrichment analysis (Figure 7) showed that the annotated DEGs were distributed in 91 metabolic pathways, which belong to 6 categories. The metabolism category contained the largest number of genes and the most secondary pathways. The secondary pathways of the metabolism categories were further analysed. This category included pathways related to the biosynthesis of other secondary metabolites, amino acid metabolism, metabolism of terpenoids and polyketides, carbohydrate metabolism, glycan biosynthesis and metabolism, lipid metabolism, signal transduction, and energy metabolism, as well as 71 other secondary pathways. Flavonoids belong to a branch of secondary metabolism. Through further analysis of tertiary pathways within the biosynthesis of other secondary metabolite pathways, it was found that the DEGs were annotated to 11 tertiary pathways within this pathway. These pathways included phenylpropanoid biosynthesis (17); flavonoid biosynthesis (10); stilbenoid, diarylheptanoid and gingerol biosynthesis (7); isoquinoline alkaloid biosynthesis (5); isoflavonoid biosynthesis (3); and other leaf-colour-related pathways (Figure 7a).

A DEG enrichment downregulation bubble diagram was constructed, which shows that the first 20 pathways enriched include pathways involving phenylpropanoid biosynthesis; stilbenoid, diarylheptanoid and gingerol biosynthesis; isoquinoline alkaloid biosynthesis; and isoflavonoid biosynthesis, as well as other pathways (Figure 7b). In conclusion, YG DEGs involved in the flavonoid synthesis pathway tended to be downregulated. Leaf colour is directly related to flavonoids, so the DEGs in these pathways regulate the synthesis and accumulation of flavonoids in *P. tatarinowii* leaves, leading to the formation of *P. tatarinowii* variegated leaves. At the same time, there were also a large number of downregulated DEGs involved in the plant hormone signal transduction (24 genes) and plant pathogen interaction (37 genes) KEGG pathways. The results showed that the different colour regions of leaves of *P. tatarinowii* also undergo great changes in expression of genes involved in the response to external stimuli and plant hormone signal transduction, which tended to be downregulated in YG.

The number of DEGs enriched in the upregulated pathways was significantly lower than that enriched in the downregulated pathways. The bubble map showed that the top 10 pathways of upregulated DEGs mainly involved oxidative phosphorylation, phagosome, glutathione metabolism, tyrosine metabolism, alpha-linolenic acid metabolism and anthocyanin biosynthesis (Figure 7c). These pathways were mainly oxidation- and metabolism-related pathways, indicating that YG presented with increased metabolism, antioxidant activity and anthocyanin synthesis.

#### 2.5.5. Flavonoid Biosynthesis Pathways in Different Leaf Colour Regions

**Figure 8:**
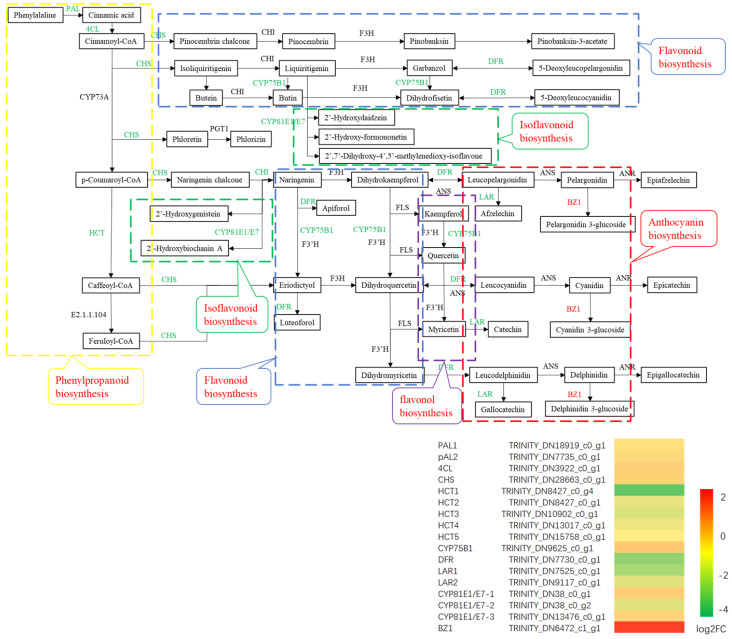
Expression of genes involved in flavonoid biosynthetic pathways in *P. tatarinowii* ‘Jinyuyuan’ leaves.

Leaf colour in plants is determined by pigments in the vacuoles, especially flavonoids and anthocyanins. Further analysis of the flavonoid synthesis pathways related to leaf colour was carried out, and a total of 32 DEGs involved in five flavonoid synthesis pathways (phenylpropanoid biosynthesis, flavonoid biosynthesis, anthocyanin biosynthesis, isoflavonoid biosynthesis, flavone and flavonol biosynthesis, 17, 10, 3, 1 and 1, respectively) were identified. The transcript abundance of eight key structural gene families, namely *PAL* (*phenylalanine ammonia-lyase*, 2 DEGs), *4CL* (*4-coumarate: coenzyme A ligase*, 1 DEG), *CHS* (*chalcone synthase*, 1 DEG), *HCT* (*shikimic acid/quinic acid hydroxy cinnamyl transferase*, 5 DEGs), *CYP75B1* (*cytochrome P450 75B1*, 1 DEG), *DFR* (*dihydroflavonol 4-reductase*, 1 DEG), *LAR* (*leucoanthocyantin reductase*, 2 DEGs) and *CYP81E1/E7* (*cytochrome P450 81E1/E7,* 3 DEGs) were lower in YG than in GR, which is consistent with the leaf colour changes (Figure 8). The *HCT*, *DFR* and *LAR* genes were significantly downregulated and only one upregulated gene (*BZ1, Bronze1*) was found, which is involved in the anthocyanin pathway. The whole flavonoid synthesis pathway was downregulated.

#### 2.5.6. Pigment- and Photosynthesis-Related Pathways

**Figure 9:**
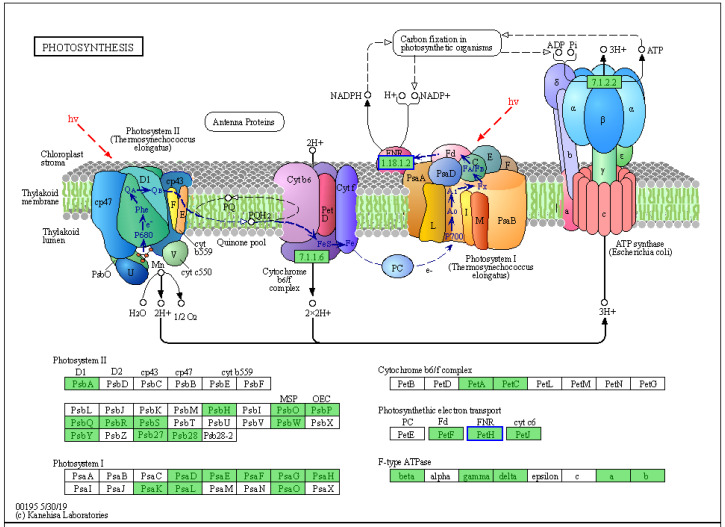
Expression of photosynthesis-pathway-related candidate DEGs. The blue boxes represent downregulated genes.

Five components, namely, photosystem II (PS-II), the cyto-chrome b6f complex, photosystem I (PS-I), the photosynthetic electron transport (PET) chain and F-type ATP synthase, work together to accomplish the light-dependent energy-producing PET reactions (Figure 9). In this experiment, the *PetH* gene encoding ferredoxin: NADP+ reductase (FNR) involved in PET was downregulated in the photosynthetic KEGG pathway, resulting in the downregulation of the FNR protein. Moreover, the downregulation of the *PetH* gene led to NADPH and ATP deficiency and then decreased photoassimilate accumulation in YG. In the passage of carbon fixation metabolism, we found that two DEGs (*maeB1* and *maeB2*) were significantly downregulated. The results showed that there were significant differences in carbon fixation, which affects carbon homogeneity, and gene downregulation weakened the fixation of CO_2_. In addition, we found that eight DEGs involved in starch and sucrose metabolism were downregulated. Downregulation of these genes may lead to a decrease in the contents of D- glucose-6P, D- glucose, a-D- glucose-1P, cellobiose, sucrose and trehalose-6P. In conclusion, the decline in photosynthetic capacity reduces the carbon assimilation and fixation to a certain extent, resulting in a reduction in photosynthetic substances and thinning of YG regions of leaves.

Chlorophyll is associated with photosynthetic components, such as PS II and PS I [27]. Photosynthetic components and chlorophyll content are interdependent. Therefore, a lack of photosynthetic components might lead to chlorophyll deficiency. In YG, only one DEG (*E3.1.1.14*) was found to be involved in the metabolic pathway of chlorophyll synthesis and degradation. This gene plays a role in the degradation of chlorophyll a and chlorophyll b into chlorophyll-esters, and downregulation of this gene may be the main reason for the lower chlorophyll content in YG compared with GR.

#### 2.5.7. Amino Acid and Lipid Metabolic Pathways

There were 16 KEGG pathways associated with amino acid metabolism identified in the transcriptome, and these pathways involved the synthesis and metabolism of several amino acids and involved taurine and hypotaurine metabolism (Table 4). There were 55 DEGs in these 16 pathways, of which only 6 DEGs were upregulated. There were 11 amino-acid-related metabolic pathways identified in the metabolome, and 18 differentially accumulated metabolites were screened, of which only two metabolites were increased, namely O-succinyl-L-homoserine (involved in cysteine and methionine metabolism) and 4-hydroxyproline (involved in arginine and proline metabolism), and the other 16 amino acid metabolites were decreased. These results indicated that the activity of the amino acid metabolic pathway of YG was downregulated, and that the abundance of corresponding amino acid products was also greatly reduced, which naturally affected the accumulation of related products and other biological activities.

Twelve lipid metabolism pathways were involved in fatty acid synthesis and degradation, sphingolipid metabolism, glycerophospholipid metabolism, steroid metabolism, etc. (Table 5). There were 27 DEGs identified as being involved in the 12 pathways, and only 2 of these DEGs were upregulated. Three of the twelve pathways were directly related to fatty acid synthesis, fatty acid elongation and fatty acid degradation. In the lipid synthesis pathway, upregulation of the *accD* (*acetyl-CoA carboxylase subunit D*) gene directly affects the synthesis of malonyl-CoA, while *FAB2* (*delta-12 fatty acid desaturase 2*) downregulation affects the production of hexadecenoic acid and octadecenoic acid. In the process of fatty acid elongation, the downregulation of the *KCS* (*3-ketoacyl-CoA synthase*) gene directly affects carbon chain elongation. Downregulation of the *ALDH* (*aldehyde dehydrogenase*) gene during fatty acid degradation affects lipid differentiation and degradation. According to the metabolome, only one alpha-linolenic acid metabolism pathway was found, with a total of two differentially accumulated metabolites, namely 9-oxononanoic acid (upregulated) and traumatic acid (downregulated). These results indicate that in YG, the lipid metabolism pathway is mostly downregulated, and the downregulation of genes affects the synthesis and metabolism of lipids, which in turn affects phospholipid bilayer formation, nucleotide generation and metabolism and other lipid-related biological activities. Indeed, the DEGs involved in purine metabolism (2) and pyrimidine metabolism (1) were downregulated at the transcriptional level.

#### 2.5.8. Computer Prediction Analysis of Transcriptome Factors (TFs)

**Figure 10:**
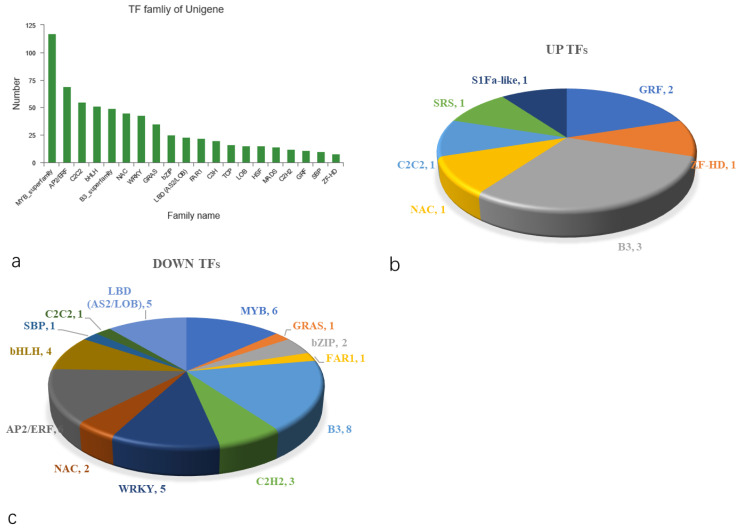
Computer prediction analysis of TFs. (**a**) Families of TF unigenes. (**b**) Upregulated differentially expressed TF genes. (**c**) Downregulated differentially expressed TF genes.

TFs can bind to cis-acting elements of target gene promoters to regulate plant development, secondary metabolism and stress resistance. Unigenes were compared to the information of the Plant Transcription Factor Database (PlantTFDB) to obtain TF family classification information corresponding to the unigenes. The prediction results of TFs revealed (Figure 10a) a total of 704 genes, belonging to 33 TF families. The top four TF families with the largest number of genes were the MYB (117), AP2/ERF (69), C2C2 (55) and bHLH (51) families. There were 55 differentially expressed TF genes, of which 10 were upregulated and 45 downregulated (Figure 10b,c). B3, AP2/ERF, MYB, WRKY and bHLH TFs were the most downregulated TFs, the numbers of which were eight, six, six, five and four, respectively.

### 2.6. RT-qPCR Validation

**Figure 11:**
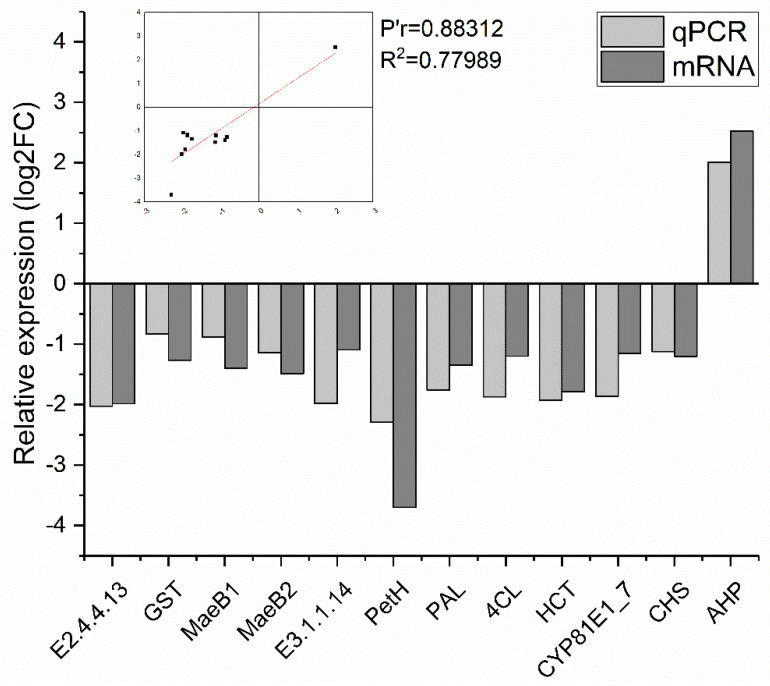
RT–qPCR verification of the expression levels of 12 DEGs identified by RNA–seq.

Twelve genes (eleven downregulated and one upregulated) that were highly and differentially expressed; that exhibited a greater than 2-fold increase in expression; and that were related to starch and sucrose metabolism, glutathione metabolism, carbon fixation in photosynthetic organisms, porphyrin metabolism, photosynthesis, flavonoid metabolism and plant hormone signal transduction were subjected to RT–qPCR. The results showed that the expression trends of all of the selected genes were consistent with those identified from the transcriptome data (Figure 11).

### 2.7. Analysis of Differentially Accumulated Metabolites

#### 2.7.1. Statistics and Analysis of Differentially Accumulated Metabolites

**Figure 12:**
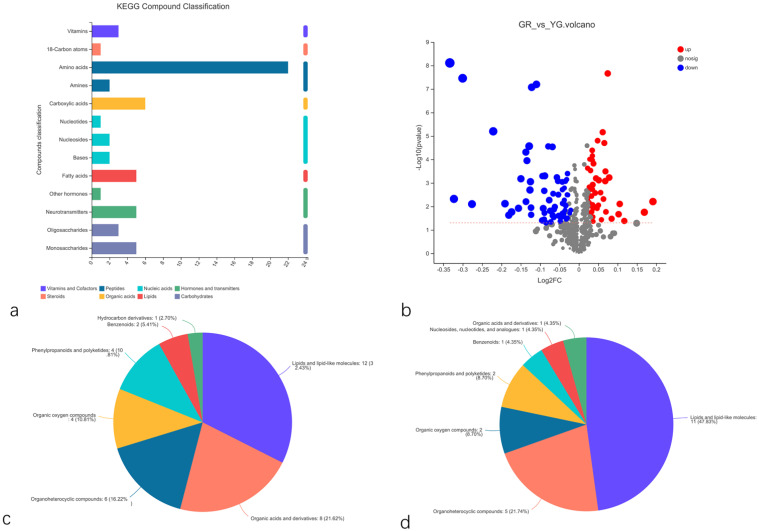
Differential metabolite analysis. (**a**) KEGG compound classification of metabolites. (**b**) Volcano plot of differentially accumulated metabolites. (**c**) Metabolites whose abundance decreased. (**d**) Metabolites whose abundance increased. GR indicates the green region of the leaf, and YG indicates the yellow–green region of the leaf.

A total of 602 metabolites were identified in the metabolome, mainly amino acids, carboxylic acids, fatty acids, neurotransmitters and monosaccharides (Figure 12a). In total, 116 differentially accumulated metabolites were detected, among which 67 decreased and 49 increased (Figure 12b). The classification of these differentially accumulated metabolites showed that the metabolites of the green tissue and yellow-green tissue were significantly different. Among the decreased metabolites, lipids and lipid-like molecules accounted for 12 metabolites, followed by organic acids and their derivatives and organoheterocyclic compounds, which accounted for 8 metabolites (Figure 12c). Conversely, lipids and lipid-like molecules (11) and organoheterocyclic compounds (5) accounted for the highest proportions of the metabolites whose abundance decreased (Figure 12d).

#### 2.7.2. KEGG Annotations of Differentially Accumulated Metabolites

**Figure 13:**
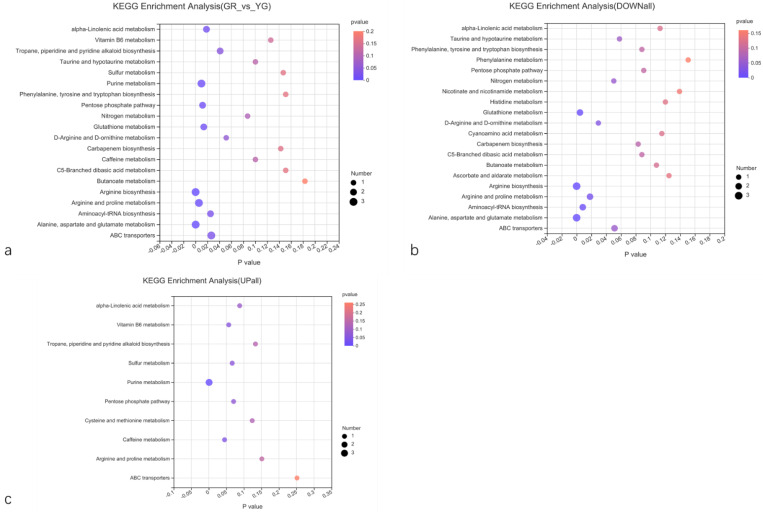
KEGG enrichment pathways of metabolites. (**a**) KEGG enrichment pathways of differentially accumulated metabolites. (**b**) KEGG enrichment pathways of downregulated differentially accumulated metabolites. (**c**) KEGG enrichment pathways of upregulated differentially accumulated metabolites. GR indicates the green region of the leaf, and YG indicates the yellow–green region of the leaf.

The differentially accumulated metabolites were enriched in 31 KEGG metabolic pathways. The differentially accumulated metabolites were involved mostly in amino acid synthesis or metabolism, purine metabolism, alpha-linolenic acid metabolism pathways, the pentose phosphate pathway and the ABC transporter pathway (Figure 13a). Significant DEGs associated with the synthesis or metabolism of multiple amino acids, pyrimidine metabolism, phenolic acids and alkaloid biosynthesis or metabolism, ascorbate and aldarate metabolism and phenylalanine metabolism were detected (Figure 13b). In addition, upregulated expression of DEGs involved in purine metabolism, caffeine metabolism and vitamin B6 metabolism was observed (Figure 13c).

### 2.8. Correlation Analysis of DEGs and Differentially Accumulated Metabolites

**Figure 14:**
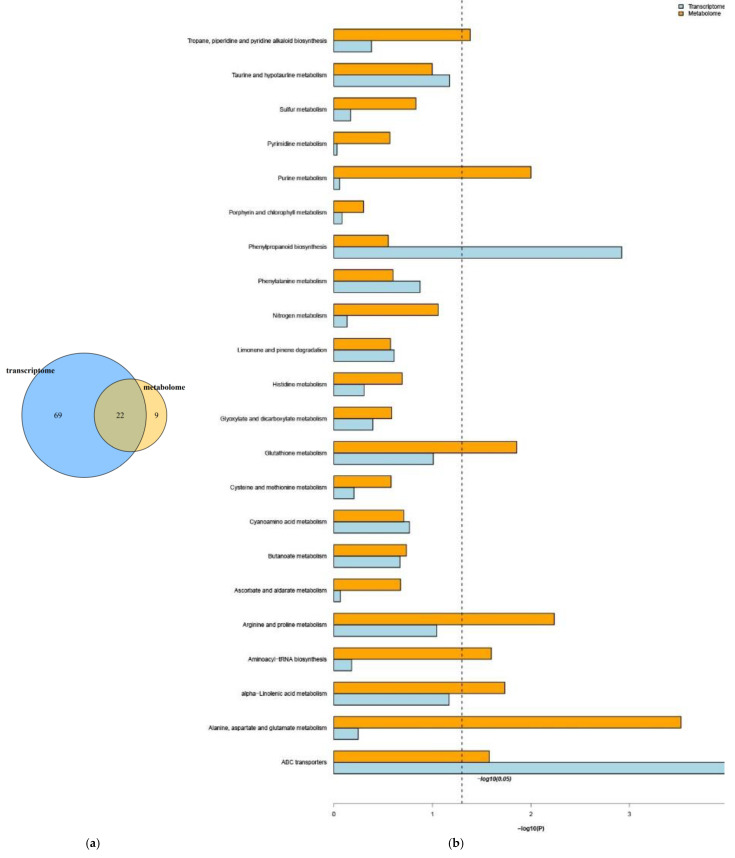
(**a**) Venn diagrams of DEGs and differentially accumulated metabolites. (**b**) KEGG enrichment pathways.

In total, 22 KEGG pathways were shared between the transcriptome and metabolome (Figure 14a). These pathways mostly focused on amino acid synthesis and metabolism, alpha-linolenic acid metabolism, phenolic acids and alkaloid biosynthesis or metabolism, ABC transporters and phenylalanine metabolism (Figure 14b). We further analysed the global metabolic pathways of the DEGs via iPath3.0 (http://pathways.embl.de (accessed on 4 August 2021)) [28]. As shown in Figure S1, most DEGs were annotated to carbohydrate metabolism, amino acid metabolism, energy metabolism, nucleotide metabolism, metabolism of cofactors and vitamins, and biosynthesis of other secondary metabolism. Among these DEGs, those involved in the following were significantly enriched: phenylalanine, tyrosine and tryptophan biosynthesis; glutathione metabolism; arginine biosynthesis; arginine and proline metabolism; alanine, aspartate and glutamate metabolism in amino acid metabolism; and purine metabolism in nucleotides. These findings indicate that there were significant differences in amino acid metabolism and nucleotide metabolism in the different colour regions of leaves of *P. tatarinowii* ‘Jinyuyuan’, which might affect the synthesis of pigments and other secondary metabolites.

## 3. Discussion

### 3.1. Explanation of Morphological Variation in P. tatarinowii ‘Jinyuyuan’ Leaves

In this study, the leaf thickness of *P. tatarinowii* ‘Jinyuyuan’ was found to differ in different leaf colour areas of the same leaf. Similar phenomena have been found in other plant species, e.g., in *H. macrophylla* ‘Maculata’ [3], *Aucuba japonica* ‘Variegata’, albino tea and *Phalaenopsis aphrodite* subsp. *formosana* [21,29,30]. In several previous studies, it has been reported that the invasion of pathogens and viruses can lead to the curling and thickness differences in leaves [31,32]. However, there are other underlying factors at play in the leaf development in this study. Paraffin sections showed that the yellow-green area of the leaves was significantly thinner than the green area; that the cells in the upper and lower epidermis, palisade tissue and spongy tissue were smaller in the yellow-green area than in the green area; and that the cells in the green area were closely arranged, while those in the yellow-green area were loosely arranged. Chloroplast ultrastructure observations revealed that the size and degree of development of chloroplasts in the yellow-green area were smaller and lower, respectively, than those in the green area, which was the direct cause of different leaf thicknesses. According to the transcriptomic and metabolic differences identified in this study, the underlying contributing factor may be that the differential expression of chlorophyll synthesis and metabolism-related genes leads to a decrease in photosynthesis levels, which leads to both a decrease in assimilation ability and a decrease in the contents of assimilates such as sucrose and starch. Guggisberg et al. [33] also showed that the abnormal xylem structure in the yellow leaf area blocked sugar transport and could not provide sufficient amounts of carbon for leaf isoprene biosynthesis through the MEP pathway. In addition, the activities of amino acid synthesis and metabolism, lipid synthesis and other metabolic pathways were obviously downregulated, and the contents of amino acids and lipids involved in differential metabolism were decreased. The decreased contents of carbohydrates, amino acids, lipids, etc., eventually lead to significantly thinner leaves in the yellow-green areas than in the green areas, resulting in different leaf thicknesses.

### 3.2. Genes Associated with Variegated Leaf Colour Formation in P. tatarinowii ‘Jinyuyuan’

Chlorophyll concentration directly affects the colour of leaves and photosynthesis. Leaf colour variation is usually caused by chlorophyll deficiency. Many chlorophyll-deficient mutants have been identified in rice, maize and *Arabidopsis thaliana* [34,35,36]. In this study, the chlorophyll content in yellow-green leaves was significantly lower than that in green areas, which affected the normal leaf colour of *P. tatarinowii* and ultimately resulted in a variegated leaf phenotype in which there was a chlorophyll deficiency. On the basis of transcriptomic differences, we identified a DEG, *E3.1.1.14*, which is related to chlorophyll synthesis and metabolism. This gene plays a role in the degradation of chlorophyll a and chlorophyll b into chlorophyllide, and its downregulation may be the main factor for the lower chlorophyll content of YG compared with GR.

The chloroplast is an important organelle for photosynthesis in plants, and it is also the site of synthesis and accumulation of photosynthetic pigments such as chlorophyll and carotenoids [37]. Studies have shown that plants with variable leaf colour also exhibit varying degrees of structural change. Yang et al. [38] reported that there was an abundance of thylakoid in the cells of *Pseudosasa japonica* green leaves, while a large number of abnormal vacuoles accumulated in albino leaf cells. Similar structural changes were also found in a study of leaf colour mutants of *Anthurium andraeanum* ‘Sonate’; chloroplasts in green leaf cells had typical characteristics, and the thylakoid membranes were clear, containing a small amount of amyloid, while the structure of granum lamellae in the mutated leaf regions was loose and scattered, with a large number of starch grains [39]. The tissue structure of yellow regions of leaves of *G. biloba* ‘Variegata’ was significantly different from that of normal green leaves. In the yellow leaves of the mutant, the chloroplast membranes appeared blurry, there were few granum lamellae layers, the lamellar structure was loose and dispersed, osmophilic particles were more distributed and densely arranged, and there were a large number of vacuoles in the cells [22]. A large amount of osmiophilic particles were also found in the white area of *H. macrophylla* ‘Maculata’ leaves [3]. In this present study, we found that ‘Jinyuyuan’ also exhibited similar phenomena: the green area of the leaves had large chloroplasts, small vacuoles, large starch grains, fewer osmophilic grains, orderly lamellae and thick lamellae of the inner capsule, while the yellow-green area had just the opposite. These structural changes may affect the development of plastids and normal photosynthesis and may hinder the synthesis and accumulation of chlorophyll, resulting in leaf colour variation.

Flavonoids are secondary metabolites of polyphenols that widely exist in various organs of plants and participate in the regulation of flower, leaf and fruit colour formation. Plant flavonoids can be divided into seven types, some of which are flavonoids, flavonols and anthocyanins [40], which are regulated by the MYB, bHLH and WD TFs [41]. Five flavonoid synthesis pathways were found in this study. We identified 32 DEGs related to flavonoid synthesis from these pathways, of which 31 were downregulated in the yellow-green region. Our RT-qPCR results also showed that the expression of genes regulating flavonoid synthesis was downregulated in the leaves of the mutant, indicating that flavonoid synthesis was inhibited in the leaves of the mutant. This may be the main reason for the yellow leaf colour of *P. tatarinowii* ‘Jinyuyuan’ leaves. In addition, the anthocyanin pathway is active in *P. tatarinowii* leaves, but leaves do not present red or blue colour, which may be due to low anthocyanin contents or the lack of expression of TF genes such as MYB, bHLH and WRKY. Studies have shown that MYB and bHLH transcription factors and WD40 proteins usually form a highly conserved MYB-bHLH-WD40 (MBW) transcription complex that regulates the biosynthesis of anthocyanins in plants [42]. In this study, 117 MYB, 51 bHLH and 43 WRKY TF genes were identified, from which 6 MYB, 5 WRKY and 4 bHLH differentially expressed TF genes were screened and removed; these genes may be closely related to leaf colour changes. It can also be inferred that there is potential to breed *P. tatarinowii* plants that have red or blue leaves.

In addition, studies have shown that plant hormones and light signals are closely related to the synthesis of flavonoids involved in flower colour and leaf colour [40,43,44]. In this study, DEGs were also enriched in signal transduction pathways, which includes plant hormone signal transduction (24 genes) and MAPK signalling pathway-plant (9 genes). Moreover, the DEGs were mostly enriched in plant hormone signal transduction (24 genes) and plant–pathogen interaction (37 genes) KEGG pathways, and there is likely coordination that occurs between the two pathways.

In conclusion, we hypothesize that the formation of variegated leaves is the direct result of transcriptional and post-transcriptional regulation. As such, we suspect that the direct cause of variegated leaves of *P. tatarinowii* ‘Jinyuyuan’ is caused by transcriptional and post-transcriptional regulation. Mutations in pigment and flavonoid synthesis pathway genes and transcription factors directly affect pigment and flavonoid synthesis and degradation, which in turn affects related protein synthesis and enzyme activity, leading to the formation of different colours.

### 3.3. Analysis of the Redox Capacity of P. tatarinowii ‘Jinyuyuan’

Aluru et al. [45] showed that oxidative stress mainly occurred in the white area of leaves, as the green area had extra energy dissipation machinery, which ultimately allowed the green area to form. Brehelin et al. [46], Velikova et al. [47] and Wang et al. [25] reported that the stromal lamellae of yellow tissue appeared blurry, that starch granules were degraded, and that plastid globules and vesicles aggregated in large numbers, all of which indicated an oxidative stress response. Similar phenomena were also found in the present study. In the yellow-green region of leaves, the chloroplasts were small, the vacuoles were large, the starch grains were small, and the osmophilic grains increased significantly in number, indicating the occurrence of oxidative stress. At the same time, the MDA content in the yellow-green region was significantly higher than that in the green region, which also reflected that the response to oxidative stress in the yellow-green region was more severe than that in the green region.

To remove reactive oxygen, cells employ a complex antioxidant system that includes antioxidant vitamins such as vitamins B, C and E, and antioxidant enzymes such as SOD, CAT and POD, which can eliminate the toxic active substances and reduce damage to cells [48,49]. In the present study, vitamin B6 in YG was significantly upregulated, and physiological indicators also showed that the activities of antioxidant enzymes in YJB were higher than those in GR; specifically, the activities of SOD, CAT and APX showed extremely significant differences in the different colour regions, indicating enhanced antioxidant capacity in the YG region. In addition, two significantly upregulated products related to amino acid metabolism were identified from the metabolomics data, one of which was 4-hydroxyproline (which is in the arginine and proline metabolism pathways), which was consistent with the proline content measurement results. The proline content in YG was significantly higher than that in GR, which also improved the tissue antioxidant capacity. At the same time, six DEGs involved in the oxidative phosphorylation pathway were identified according to the transcriptomic data and the upregulated expression of four of these DEGs in turn regulated the decomposition of peroxide into H_2_O, eliminating toxic substances and reducing damage to cells, and two downregulated DEGs were found to regulate H^+^ production (Figure S2). The mechanism of enhanced antioxidant activity in the YG region was analysed at the gene level.

## 4. Materials and Methods

### 4.1. Plant Materials

The experimental materials used were three-year-old *P. tatarinowii* ‘Jinyuyuan’ grafted seedlings. The test site was located in the city of Tai’an, Shandong Province (36°11′07′′ N, 117°06′51′′ E). Young leaves of ‘Jinyuyuan’ were collected in May 2021, and the GR and YG in each of the used leaves were separated. The leaves were wrapped in aluminium foil and immediately flash frozen in liquid nitrogen, after which samples were brought back to the laboratory and stored at −80 ℃. Each sample contained more than 10 leaves.

### 4.2. Morphological Observations

Leaves were assessed with a Royal Horticultural Society Colorimetric Card (RHSCC) under natural light. RHSCC is published by the Royal Horticultural Society and is the standard reference for recording plant colour in horticulture worldwide, with 920 colours. The plant part was placed under the colorimetric hole or next to the colour block. A portable chroma-meter (CM-5, Konica Minolta, Japan) was used to measure the brightness (L*) and two chroma components (a * and b *) in different colour areas of the leaves under natural light, which was repeated 5 times. The chroma (c*) and phase angle (h) were then calculated as c* = (a*2 + b*2)^1/2^; h = arctan (a*/b*).

### 4.3. Microstructure and Ultramicrostructure Observations

Leaves including both green and yellow-green zones were collected, fixed in formalin-acetic acid-alcohol (FAA) for 12 h with a vacuum device and then subjected to a gradient of alcohol dehydration. The treated leaves were subsequently embedded in paraffin and cut into 10 μm slices with an RM2016 slicer (Leica, Wetzlar, Germany). After dewaxing, the samples were stained with 1% sarranine and 0.5% fast green and then incubated with three tubes of xylene for 5 min. Finally, the tissue sections were mounted with neutral balsam. Images were then collected with an upright optical microscope equipped with a digital camera (Eclipse E100+DS U3) (Nikon, Tokyo, Japan) [50].

Pieces (1 mm × 1 mm) of the green and yellow-green regions of the leaves were fixed in 2.5% glutaraldehyde (pH 7.4) for 2 h with a vacuum device. After washing three times with 0.1 M phosphate buffer (pH 7.2), the cells were fixed in 1% osmic acid at 4 °C for 2 h. Then, the samples were dehydrated via a gradient of ethanol. Subsequently, the samples were embedded in Epon-Araldite resin for penetration and placed in a mould for polymerization. After semithin sections were used for positioning, ultrathin sections of 60–80 nm were made using a Leica UC7 ultrathin slicer (Leica, Wetzlar, Germany) and collected for microstructure analysis. The cells were subsequently counterstained with 3% uranyl acetate and 2.7% lead citrate. Then, the cells were observed with an HT7700 transmission electron microscope (Hitachi, Tokyo, Japan) [51].

### 4.4. Determination of Pigment Contents

For determination of the chlorophyll content, a plant chlorophyll content kit (CPL-2-G, Comin Biotechnology, Suzhou, China) was used. The chlorophyll a content (mg/g fresh weight) was calculated as 0.01*(12.7*A663-2.69*A645)*D/m; the chlorophyll b content (mg/g fresh weight) was calculated as 0.01*(22.9*A645-4.68*A663)*D/m; and the total chlorophyll content (mg/g fresh weight) was calculated as 0.01*(20.21*A645 + 8.02*A663)*D/m, where D is the dilution factor and m is the sample mass (g). The carotenoid content (μg/g fresh weight) was calculated as 31.25 × ΔA/W, where W is the sample quantity (g); ΔA is equal to the A of the determination tube, the A of a blank tube, for which a spectrophotometer was used to quickly determine the absorbance value at 450 nm (A).

### 4.5. Assays of Protective Enzyme Activities and MDA Contents

Samples (0.1 g) of the GR and YG were separately weighed and ground in 1 mL of extraction solution in an ice bath. Then, the homogenate was poured into a centrifuge tube and centrifuged at 8000× *g* for 10 min at 4 °C. The soluble protein content was measured using a BCAP-1-W assay kit (Comin, Suzhou, China). The activities of SOD, POD, CAT and APX were measured using a BC0170 SOD assay kit (Comin, Suzhou, China), a BC0090 POD assay kit (Comin, Suzhou, China), a BC0200 CAT assay kit (Comin, Suzhou, China) and an APX-1-W assay kit (Comin, Suzhou, China). Similarly, the MDA content was measured using a BC0025 MDA assay kit (Comin, Suzhou, China) [52,53]. Assays were performed according to the manufacturer’s instructions from the Suzhou Cominbio Technology Co., Ltd. (http://www.cominbio.com/ (accessed on 4 August 2021)).

### 4.6. RNA Preparation, RNA Sequencing (RNA-seq) and Differential Gene Expression Analysis

The GR and YG leaf tissue (100 mg) from frozen leaf tissue (3 replicates) was ground through a high-throughput grinder (wonbio-96, Shanghai, China), which was placed in the grinding tube (including a 0.6 cm steel ball). The grinding tube and the metal adapter were immersed in liquid nitrogen and frozen, and the grinding was carried out at 55 Hz and 15 s with high-speed shock grinding. Total RNA was extracted from samples using TRIzol^®^ reagent (an RNA purification reagent for plant tissue) according to the manufacturer’s instructions (Invitrogen, Carlsbad, CA, USA), and genomic DNA was removed using DNase I (Takara Biomedical Technology (Beijing) Co., Ltd., Beijing, China). Then, the integrity and purity of the total RNA quality were determined by a 2100 Bioanalyzer (Agilent Technologies, Inc., Santa Clara, CA, USA) and quantified using an ND-2000 instrument (NanoDrop, Thermo Scientific, Wilmington, DE, USA). Only high-quality RNA samples (OD260/280 = 1.8~2.2, OD260/230 ≥ 2.0, RNA (1 μg) integrity number (RIN ≥ 8.0, 28S/18S ≥ 1.0)) were used to construct sequencing libraries. Oligo (dT) Magnetic beads were used for A–T base pairing with polyadenylation to isolate mRNA from total RNA for analysis of transcriptome information. The cDNA libraries were sequenced via paired-end sequencing using the Illumina NovaSeq 6000 System platform (Illumina, CA, USA). We used DESeq2software to determine the statistical enrichment of DEGs in Gene Ontology (GO) and Kyoto Encyclopedia of Genes and Genomes (KEGG) pathways [54]. 

### 4.7. RT-qPCR Validation

RNA was used for cDNA synthesis using a reverse transcription kit (Takara). Quantitative PCR was performed on a 7500 Fast Real-Time PCR System (Thermo Fisher Scientific) using a TB Green^®^ Premix Ex Taq™ Kit (TaKara). The amplification and extension rate of this Taq enzyme was 1–2 kb/min, and the fluorescence quantitative extension time was 30 s, which can cover a length of approximately 500 bp. The PCR procedure was as follows: holding at 95 °C for 30 s, followed by 40 cycles of 95 ℃ for 5 s and then melting at 60 °C for 30 s. Transcription initiation factor (*TAF*) was used as an internal control for RT-qPCR analysis. By the use of the 2^−ΔΔT^ method, the relative expression levels of candidate genes were obtained by normalization with *TAF* expression, and each target sample was biologically replicated three times. All the values represent the means ± SDs (*n* = 3), and Student’s t-test was used to determine significant differences. The sequences of all primers used are listed in Table S4.

### 4.8. UPLC-MS

Fifty milligrams of solid leaf material were accurately weighed, and the metabolites were extracted using 400 µL of methanol/water (4/1, *v*/*v*) solution. The mixture was then incubated at −20 °C and subjected to a high-throughput tissue crusher (Wonbio-96c, Shanghai Wanbo Biotechnology Co., Ltd., Shanghai, China) at 50 Hz for 6 min, followed by vortexing for 30 s and ultrasonication at 40 kHz for 30 min at 5 °C. The samples were then incubated at −20 °C for 30 min to precipitate the proteins. After centrifugation at 13,000× *g* at 4 ℃ for 15 min, the supernatant was carefully transferred to sample vials for LC-MS/MS (UHPLC -Q Exactive HF-X, Thermo Fisher Technology (Shanghai, China) Co., Ltd.).

### 4.9. Data Analysis

The data were statistically analysed, and data processing and analysis were performed with Excel 2010 and SPSS 22.0 statistical analysis software, respectively. Analysis of variance was performed to determine the differences in the parameters between the two different kinds of leaf tissues. For RNA-seq, statistical analysis of the raw counts of genes that were differentially expressed in the two conditions was performed with DESeq2 software. Genes with a false discovery rate (FDR) < 0.01 and a fold change (FC) ≥2 according to DESeq analysis were considered differentially expressed. For UPLC-MS/MS, information for chemical compounds was obtained from mass spectra, retention times, the Human Metabolome Database (HMDB) (http://www.hmdb.ca/ (accessed on 4 August 2021)), the METLIN (https://metlin.scripps.edu/ (accessed on 4 August 2021)) database and a custom-made database, and the resulting data were analysed by principal component analysis and t-tests. Hierarchical cluster analysis (HCA) and orthogonal partial least-squares discriminant analysis (OPLS-DA) were performed by R-software to identify the differences between the nine leaf samples. OPLS-DA was used to identify the differences in metabolite composition between the samples according to variable importance in projection (VIP) values ≥ 1 and fold changes ≥2 or ≤0.5.

## 5. Conclusions

Unbalanced development led to different colour regions of leaves of *P. tatarinowii* ‘Jinyuyuan’. The yellow-green regions of the leaves were significantly thinner than the green regions were. The chloroplast ultrastructure showed that the chloroplasts in the yellow-green regions were small, the volume of the vacuoles was large, the volume of the starch grains was small, the number of osmophilic grains increased significantly, the lamellae of the inner member were loosely arranged and thin (which was accompanied by oxidative stress), and the antioxidant activity increased. The transcript levels in the yellow-green region were mostly decreased, in which 31 differentially downregulated genes were identified, and only 1 differentially upregulated gene involved in the flavonoid synthesis pathway was detected. In the yellow-green regions, a key gene involved in chlorophyll synthesis and metabolism was downregulated, which led to significantly less chlorophyll in YG than in GR. In addition, the photosynthetic level in YG decreased, which led to a decrease in assimilation ability; reduced sucrose, starch and other assimilate contents; and obviously thinner leaves. In addition, the activity of the amino acid synthesis and metabolic pathways and lipid synthesis and metabolic pathways showed an obvious downward trend, and the contents of amino acids and lipids decreased; however, the oxidative phosphorylation pathway related to oxidative activity showed an upward trend, the vitamin B6 content increased, and the antioxidant capacity of cells in the yellow-green area showed an upward trend. In summary, the variegated leaves of *P. tatarinowii* ‘Jinyuyuan’ are the result of transcriptional and post-transcriptional regulation. Mutations in genes and TF genes involved in the pigment and flavonoid synthesis pathways directly affect the pigment and flavonoid synthesis and degradation, further affecting the carbon assimilation and carbon fixation related to protein synthesis and enzyme activity, lipid synthesis and degradation, and other metabolic pathways. Thus, the plant leaves present a different colour.

## Figures and Tables

**Table 1:** Results of leaf colour determination.

	GR	YG
L*	47.70 ± 0.70 ^B^	56.70 ± 1.17 ^A^
a*	−7.68 ± 1.33	−7.14 ± 0.73
b*	−5.46 ± 0.86 ^B^	4.80 ± 1.11 ^A^
c*	9.50 ± 0.76	8.65 ± 0.82
h	35.78 ± 8.45 ^A^	−33.72 ± 6.72 ^B^
RHSCC	137B	N144D

Note: L* stands for the brightness, and a* and b* stand for two chroma components in different colour areas of the leaves under natural light; the c* (chroma) and h (phase angle) were then calculated as c* = (a*2 + b*2) 1/2; h = arctan (a*/b*). GR indicates the green region of the leaf, and YG indicates the yellow-green region of the leaf. RHSCC is the Royal Horticultural Society Colorimetric Card. The 137B and N144D are colour groups, and the uppercase letters indicate very significant differences.

**Table 2:** Anatomic characteristics of different parts of leaves of *P. tatarinowii* ‘Jinyuyuan’.

Type	Upper Epidermis Thickness (μm)	Lower Epidermis Thickness (μm)	Leaf Thickness (μm)	Palisade Cells Thickness (μm)	Spongy Cells Thickness (μm)	Palisade Cells/Spongy Cells
GR	8.580 ± 2.614	7.029 ± 1.668	105.585 ± 6.924 ^A^	44.626 ± 8.310	48.660 ± 7.197 ^a^	0.953 ± 0.316
YG	8.487 ± 1.569	6.218 ± 1.509	84.288 ± 7.815 ^B^	37.644 ± 6.918	35.967 ± 7.809 ^b^	1.073 ± 0.219

Note: The uppercase letters indicate very significant differences; the lowercase letters indicate significant differences. GR indicates for the green region of the leaf, and YG indicates the yellow-green region of the leaf.

**Table 3:** Pigment contents in different parts of leaves of *P. tatarinowii* ‘Jinyuyuan’.

	GR	YG
Chl a (mg g^−^^1^ FW)	0.40 ± 0.0021 ^A^	0.31 ± 0.0003 ^B^
Chl b (mg g^−^^1^ FW)	0.19 ± 0.0026 ^A^	0.15 ± 0.0024 ^B^
Chl a + b (mg g^−^^1^ FW)	0.59 ± 0.0032 ^A^	0.47 ± 0.0028 ^B^
Chl a/b	2.14 ± 0.0320 ^a^	2.06 ± 0.0307 ^b^
Car (ug g^−1^ FW)	118.12 ± 0.0307 ^A^	110.50 ± 0.0178 ^B^
Chl a + b /Car	4.97 ± 0.0269 ^A^	4.22 ± 0.0244 ^B^

Note: Different capital letters indicate significant differences at 0.01 level, and different lowercase letters indicate significant differences at 0.05 level. GR indicates for the green region of the leaf, and YG indicates the yellow-green region of the leaf.

**Table 4:** Amino acid metabolic pathways.

Transcriptome				Metabolomic			
Pathway ID	Description	Number		Pathway ID	Description	Number	
		Down	Up			Down	Up
map00250	Alanine, aspartate and glutamate metabolism	2	0	map00220	Arginine biosynthesis	3	
map00260	Glycine, serine and threonine metabolism	2	1	map00250	Alanine, aspartate and glutamate metabolism	3	
map00270	Cysteine and methionine metabolism	4	0	map00270	Cysteine and methionine metabolism		1
map00280	Valine, leucine and isoleucine degradation	1	1	map00330	Arginine and proline metabolism	2	1
map00300	Lysine biosynthesis	2	0	map00340	Histidine metabolism	1	
map00310	Lysine degradation	1	0	map00360	Phenylalanine metabolism	1	
map00330	Arginine and proline metabolism	5	0	map00400	Phenylalanine, tyrosine and tryptophan biosynthesis	1	
map00340	Histidine metabolism	1	0	map00430	Taurine and hypotaurine metabolism	1	
map00350	Tyrosine metabolism	4	1	map00460	Cyanoamino acid metabolism	1	
map00360	Phenylalanine metabolism	4	1	map00472	D-Arginine and D-ornithine metabolism	1	
map00380	Tryptophan metabolism	7	0	map00480	Glutathione metabolism	2	
map00410	beta-Alanine metabolism	3	1				
map00430	Taurine and hypotaurine metabolism	2	0				
map00450	Selenocompound metabolism	1	0				
map00460	Cyanoamino acid metabolism	5	0				
map00480	Glutathione metabolism	5	1				

**Table 5:** Lipid metabolic pathways.

	Pathway ID	Description	Number	
			Down	Up
Transcriptome	map00061	Fatty acid biosynthesis	1	1
	map00062	Fatty acid elongation	2	0
	map00071	Fatty acid degradation	1	
	map00073	Cutin, suberine and wax biosynthesis	1	
	map00100	Steroid biosynthesis	1	
	map00561	Glycerolipid metabolism	6	0
	map00564	Glycerophospholipid metabolism	3	
	map00590	Arachidonic acid metabolism	1	
	map00591	Linoleic acid metabolism	2	0
	map00592	alpha-Linolenic acid metabolism	4	1
	map00600	Sphingolipid metabolism	2	0
	map01040	Biosynthesis of unsaturated fatty acids	1	
Metabolomic	map00592	alpha-Linolenic acid metabolism	1	1

## Data Availability

Transcriptome sequencing data and metabolomic data are available in the Majorbio cloud platform website (https://cloud.majorbio.com/page/project/p.html (accessed on 4 August 2021)) under the accession number MJ20210616098-FX2021080400095 and MJ20210728070.

## Supplementary Materials

The following supporting information can be downloaded at: https://www.mdpi.com/article/10.3390/ijms23094950/s1.
