# Supplementary material for: Comparative Analysis of the Metabolome and Transcriptome between the Green and Yellow-Green Regions of Variegated Leaves in a Mutant Variety of the Tree Species Pteroceltis tatarinowii
Source: Int J Mol Sci. 2022 Apr 29;23(9):4950. doi: 10.3390/ijms23094950 (PMC9101679; doi:10.3390/ijms23094950)
Supplement: Supplementary file 1 [file ijms-23-04950-s001.zip › ijms-1665973-supplementary.pdf]

Table S1. Statistics of output data of six samples.

Sample	Raw reads	Raw bases	Clean reads	Clean bases	Error rate (%)	Q20(%)	Q30(%)	GC content (%)
GR1	43955710	6637312210	43631114	6485332563	0.0246	98.21	94.46	45.52
GR2	43253262	6531242562	42949114	6381978387	0.0248	98.14	94.26	45.17
GR3	42955164	6486229764	42633900	6340489258	0.0246	98.21	94.48	44.70
YG1	44365454	6699183554	43951406	6536035110	0.0246	98.19	94.54	45.09
YG2	52296666	7896796566	51890274	7700528119	0.0245	98.24	94.55	45.32
YG3	41094562	6205278862	40772644	6058882218	0.0246	98.21	94.44	44.93

Note: GR indicates for the green region of the leaf, and YG indicates the yellow-green region of the leaf. Raw reads: the number of reads in the raw data; Raw bases: the total number of bases in the raw data .Clean reads: the number of high-quality reads after filtering the raw data; Clean Bases: the total number of bases in high-quality reads; Error rate: the overall sequencing error rate; Q20: the percentage of bases with a Qphred value of not less than 20 to the total number of bases; Q30: the percentage of bases with a Qphred value of greater than 30 to the total number of bases; GC Content: the sum of the number of G and C in high-quality reads is the percentage of the total number of bases.

Table S2. Statistics of the spliced transcriptome.

Type	Unigene	Transcript
Total number	41833	80323
Total base	47941007	1.14E+08
Largest length (bp)	13639	13639
Smallest length (bp)	201	201
Average length (bp)	1146.01	1424.1
N50 length (bp)	2154	2161
E90N50 length (bp)	2728	2297
Fragment mapped percent (%)	61.409	76.463
GC percent (%)	38.93	39.22
TransRate score	0.29255	0.36925
BUSCO score	C:68.1%[S:66.7%;D:1.4%]	C:90.9%[S:50.9%;D:40.0%]

Note: GR indicates for the green region of the leaf, and YG indicates the yellow-green region of the leaf.

Table S3. Statistics of transcriptome annotation results.

	Exp_Unigene number(percent)	Exp_Transcript number(percent)	All_Unigene number(percent)	All_Transcript number(percent)
GO	18848(0.4614)	47847(0.6083)	19002(0.4542)	48559(0.6045)
KEGG	9405(0.2302)	26448(0.3363)	9460(0.2261)	26852(0.3343)
COG	18143(0.4442)	47724(0.6068)	18257(0.4364)	48402(0.6026)
NR	22520(0.5513)	56366(0.7166)	22701(0.5427)	57168(0.7117)
Swiss-Prot	16965(0.4153)	44156(0.5614)	17064(0.4079)	44797(0.5577)
Pfam	17030(0.4169)	43374(0.5515)	17112(0.4091)	43972(0.5474)
Total_anno	22736(0.5566)	56655(0.7203)	22926(0.548)	57467(0.7154)

Note: GO: Gene Ontology; KEGG: Kyoto Encyclopedia of Genes and Genomes; COG: Cluster of Orthologous Groups of proteins; NR: NCBI non-redundant protein sequences; Swiss-Prot: a

manually annotated and reviewed protein sequence database;
Pfam: Protein families detabase.

Table S4. Primer sequences of target genes.

Gene	Description	Gene ID	Sequence (5′->3′)	Length (bp)
E2.4.1.13	Sucrose synthase	TRINITY_DN8933_c0_g1	F: ACCGCCACCTCTCTGCTAAAC<br>R: CTCATTCTCCAATGCACGAAC	467
GST	Glutathione S-transferase	TRINITY_DN827_c0_g2	F: CAGATGAAGAGGGAAAGAAAGC<br>R: GGATAACACCACTAACAGCAGGG	270
MaeB 1	Malic oxidoreductase	TRINITY_DN4277_c0_g1	F: GGCACTCACATTTTCCATTTAGTT<br>R: CCAGATCCTCTTTCGTGTCTCTTC	243
MaeB 2	Malic oxidoreductase	TRINITY_DN7682_c0_g1	F: AATTGGAACATCCGGAGTAGG<br>R: CTTTAGGCTGAGGAAGGCGAG	473
E3.1.1.14	Chlorophyllase	TRINITY_DN5431_c0_g1	F: CACGAAACCAAGCCTCACATC<br>R: ACCATAGCCCTTAGCCACAAA	198
PetH	encoding ferredoxin--NADP reductase	TRINITY_DN10087_c0_g2	F: CTACTGGGACTGGGGTTGCTC<br>R: CTTTAAGGCGTTTTCGATCGG	332
PAL	Phenylalanine ammonia-lyase	TRINITY_DN7735_c0_g1	F: TGAGGCATTTGGAGGAGAATTT<br>R: TTTTGCATCAGCGGATAGGTA	203
4CL	4-coumarate CoA ligase	TRINITY_DN3922_c0_g1	F: TCCCTCTCCACACTTACTGCTTC<br>R: GGTATGTCTTTCTCATTGGCCTC	463
HCT	Transferase	TRINITY_DN13017_c0_g1	F: TCCTAACCCTTTTTTACCCTCTG<br>R: CCTCTTTGCTATTACGTTTCTCG	434
CYP81E1_7	Cytochrome P450	TRINITY_DN38_c0_g1	F: TCGAGATCAGAAGAGACGAGATAA<br>R: TGAGACTCCTGCAAAGAGAGTAAA	409
CHS	Chalcone synthase	TRINITY_DN28663_c0_g1	F: CCAAGGCCAAGATCACCCAC<br>R: TCTCCGAGCAAACGACCAAT	219
AHP	histidine phosphotransferase	TRINITY_DN8549_c0_g1	F: CTCCAAGATGATGCTAATCCAAAC<br>R: TTGTGCTACTTCCCTTCAACTGAT	163
TAF	Transcription initiation factor	TRINITY_DN3950_c0_g1_i2	F: TGGAAACAGGCAAGTCAGAAAG<br>R: GAAGAATCGTCGGAGTGGAGAG	148

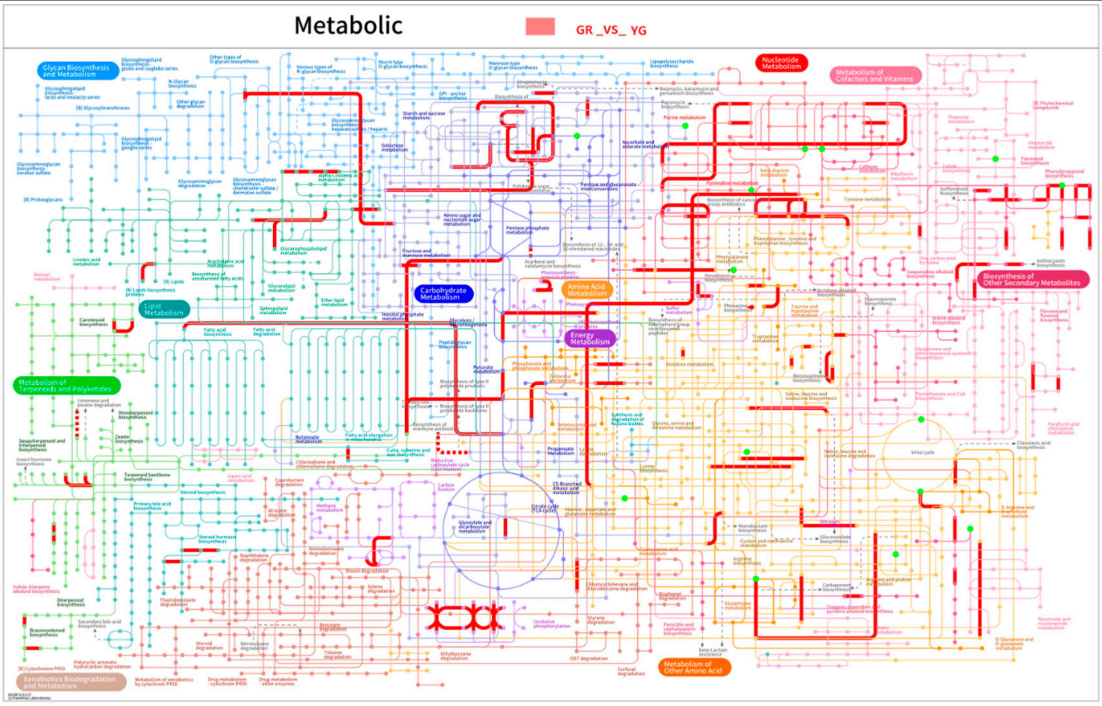

Figure S1. Diagram of iPath integrated analysis. GR indicates for the green region of the leaf, and YG indicates the yellow-green region of the leaf.

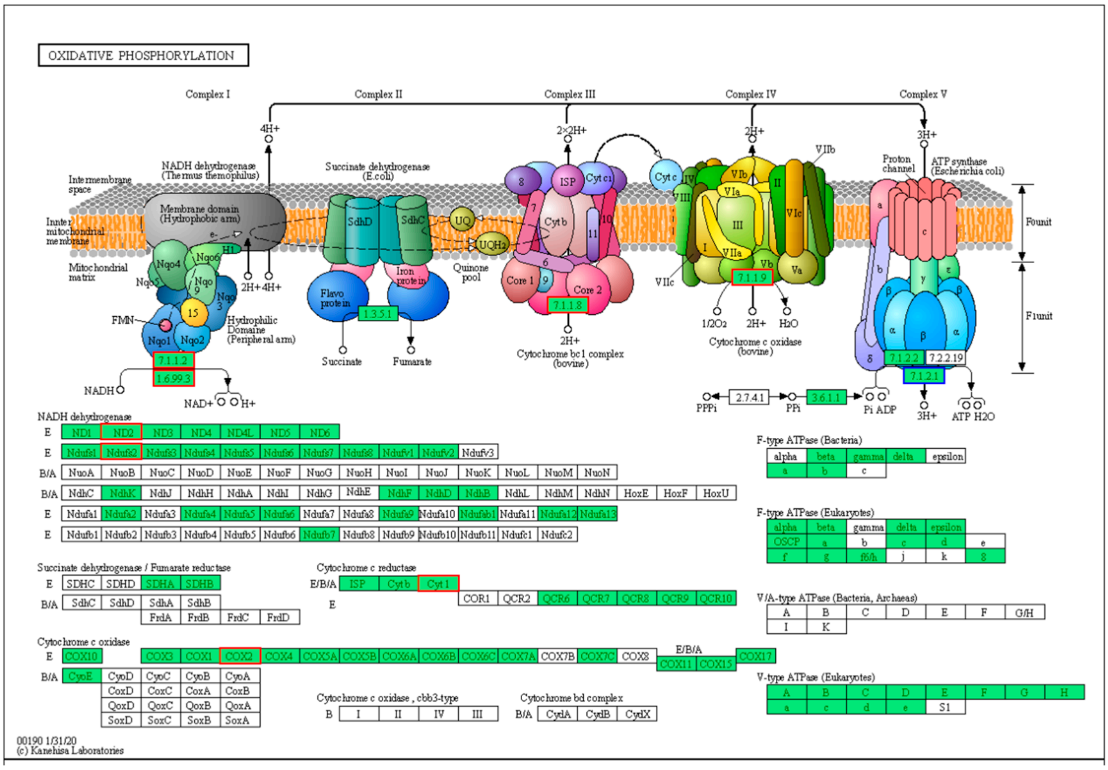

Figure S2. Oxidative phosphorylation pathway.
